# Supplementary material for: Patterns of Intron Gain and Loss in Fungi
Source: PLoS Biol. 2004 Nov 30;2(12):e422. doi: 10.1371/journal.pbio.0020422 (PMC532390; doi:10.1371/journal.pbio.0020422)
Supplement: Table S1 — Also available at http://genes.mit.edu/NielsenEtAl/. (4.3 MB ZIP). [file pbio.0020422.st001.zip › NielsenEtAl/html/745.html]

AN0154.1.NCU08729.1.MG09574.1.FG01623.1


```
 CLUSTAL W (1.82) Multiple Sequence Alignments - Introns Inserted


Sequence 1: NCU08729.1	245 aa
Sequence 2: FG01623.1	190 aa
Sequence 3: MG09574.1	208 aa
Sequence 4: AN0154.1	268 aa
Alignment Length: 275 aa
Number Identitical Residues: 100 aa
Alignment Score (without introns) 4549


MG09574.1 	-----------------------------------------------MATNQPPEIPAEQ
NCU08729.1	MSTIPPPQGNGLPSQPPAGATAPAAAAAP--------AAADAATMEDQAAADGATTTAAA
FG01623.1 	------------------------------------------------MASDAPEKTAEQ
AN0154.1  	MSDTKPPSTLPQSQPPPNSQDQSSEAIVVPKLEPDSDATALDTSIEQEAVMAGPSVNSAA
          	 :   ...    .. .. .   .: :   .. ...:.:::  :: ..  .   .   :  

MG09574.1 	----------PPAASQTAGEQQPEEEQPAPLPEPKAVTRKDIPLKDFLAKMDDYAPI0IP
NCU08729.1	GGDATANTTTTTTAPAAANDDQRPNMDIPSLPEPKLPTRKDVSLKELLSKMDEYAPI0IP
FG01623.1 	----------TPPAPEVPSEEAPP---APTVPESRLPTRKDVSLREFLNKMDDYAPI0IP
AN0154.1  	AGPDAG----AAPGAGVGDDQIGNPVQAPTSVDAAATSKKETSLREFLGKMDDYAPI0IP
          	..  :.    ..... . .::     . ..  :.   ::*: .*:::* ***:**** **

MG09574.1 	DAVTNYYMTRAGLPPP---P-QTDQRLARLLALATQKFIADIAADAYQYSRIRASNSSAS
NCU08729.1	DAVTSYYMTRAGLPPP---P-QTDQRLARLLALATQKFIADIAADAYQYSRIRASN-TNA
FG01623.1 	DAVTNYYMTKAGLPPP---P-QTDPRLARLLALATQKFIADIAADAYQYSRIRAS--SNT
AN0154.1  	DAVTAHYLTLAGLPPPGHGPNQTPPHLARLLALATQKFIADIAADSYQYARIRASNSSSA
          	**** :*:* ******. .*.**  :*******************:***:*****.:: :

MG09574.1 	NNPMGHLGAAAGFPVPGQPGGVQGGAGGAGGGGGAGGKDAGRGPP-----LGVQRPGYGG
NCU08729.1	NNPMGSLGAAAGFPIPGQP-----------TNQAAGAKDQGRGGP-----LGIQRPGYGG
FG01623.1 	NNPMGSLGAAAGFPIPGQP-----------TGQP-GSKDQTKGAP-----LGIQRPGYGG
AN0154.1  	SNPMGSLNAASGLGLAGAGG---AGAGSAGGAGTGGAGETGKGGSKAGTHLGIQRPGFGG
          	.**** *.**:*: :.*  .   ..:..:.    .*. :  :* ..:.: **:****:**

MG09574.1 	GGQGGG-QNRTVLTMEDLGMAVGEYGVNVKRSEFYR
NCU08729.1	GGQGGS-QNRTVLTMEDLGMAAGEFGVNVKRSEFYR
FG01623.1 	GGQGGS-QNRTVLTMEDLGMAVGEYGVNVKRSEFYR
AN0154.1  	GGSGGSGQGRTVLTMEDLGMAVAEYGVSVKRGEFYR
          	**.**..*.************..*:**.***.****
```
